# IGH::FGFR3和IGH::CCND1并存的多发性骨髓瘤2例报告并文献复习

**DOI:** 10.3760/cma.j.cn121090-20250421-00195

**Published:** 2026-01

**Authors:** 亚楠 常, 雅蓉 王, 琦 孙, 志坚 肖, 成华 崔

**Affiliations:** 1 中国医学科学院血液病医院（中国医学科学院血液学研究所），血液与健康全国重点实验室，国家血液系统疾病临床医学研究中心，细胞生态海河实验室，天津 300020 State Key Laboratory of Experimental Hematology, National Clinical Research Center for Blood Diseases, Haihe Laboratory of Cell Ecosystem, Institute of Hematology & Blood Diseases Hospital, Chinese Academy of Medical Sciences & Peking Union Medical College, Tianjin 300020, China; 2 天津医学健康研究院，天津 301600 Tianjin Institutes of Health Science, Tianjin 301600, China

## Abstract

回顾性分析2例携带IGH::FGFR3与IGH::CCND1的多发性骨髓瘤（MM）患者的临床资料，分析其克隆模式，并对相关文献进行复习。FISH检测显示2例患者均存在双等位IGH易位。例1为冒烟型MM，91％的骨髓瘤细胞呈现双IGH基因易位共阳性；例2为活动性MM，骨髓瘤细胞中93％显示IGH::FGFR3单阳性信号，另可见5％合并IGH::CCND1。本研究显示双等位IGH原发性易位导致IGH::FGFR3和IGH::CCND1共存，揭示了初发克隆IGH双等位基因易位驱动的新型遗传学模式，同时阐明了双等位基因易位存在克隆异质性。

## Abstract (en)

We retrospectively analyzed the clinical data and clonal architecture of two multiple myeloma（MM）patients harboring concurrent IGH::FGFR3 and IGH::CCND1 fusions, with a review of relevant literature. Fluorescence in situ hybridization（FISH）confirmed biallelic IGH translocations in both cases. Case 1, diagnosed with smoldering MM（SMM）, exhibited co-positivity for both IGH translocations in 91％ of bone marrow plasma cells. Case 2, with active MM, showed predominant IGH::FGFR3 positivity（93％ of cells）, with a minor subclone（5％）co-expressing IGH::CCND1. This study demonstrates that primary biallelic IGH translocations can lead to the coexistence of IGH::FGFR3 and IGH::CCND1, revealing a novel genetic mechanism driven by biallelic IGH translocations in the founding clone. Furthermore, it elucidates the clonal heterogeneity in biallelic IGH translocation events.

多发性骨髓瘤（MM）是浆细胞恶性增殖性疾病，其发病机制涉及复杂的遗传学异常[1]。IGH易位通常为MM的早期遗传学事件，目前已鉴定出4类特征性初级伙伴基因：t（4;14）、t（11;14）、t（14;16）及t（14;20），分别介导NSD2/FGFR3、CCND1、MAF和MAFB基因的异常激活[2]–[3]。此外，MM的继发遗传学异常中常见“二次IGH易位”，其发生率随着疾病分期的进展而增加，多为涉及MYC基因的t（8;14）易位。特别需要指出的是，同一肿瘤细胞中同时存在两种初级IGH易位的现象在早期尤为罕见。本研究显示了MM患者中IGH::CCND1和IGH::FGFR3易位共存的现象，并结合既往文献探讨其实验室特征及临床意义。

## 病例资料

例1，男，66岁，主因“泡沫尿1年余”于2025年2月就诊于我院。患者2023年12月因泡沫尿就诊于当地医院，尿蛋白85 mg/24 h。2024年6月复查尿蛋白115 mg/24 h，肌酐正常。2024年12月复查尿蛋白500 mg/24 h，肌酐仍正常。就诊于肾病中心，血清免疫固定电泳示IgG λ阳性；血清游离轻链比值（游离κ轻链∶游离λ轻链）0.05。骨髓涂片：浆细胞占18.5％；骨髓免疫分型：异常浆细胞占13.2％。骨髓活检：异常浆细胞占20％；FISH：20％细胞1q21扩增。诊断考虑为冒烟型MM（SMM）。为进一步评估病情及治疗于我院就诊，患者入院后完善相关检查。实验室检查显示IgG λ型M蛋白血症（血清M蛋白21.54 g/L，尿M蛋白1 023 mg/24 h）。骨髓涂片浆细胞17.5％，骨髓活检示20％～30％浸润；外周血未见浆细胞。HGB 159 g/L，白蛋白41.01 g/L，肌酐88.26 µmol/L，血钙2.44 mmol/L，乳酸脱氢酶175.38 U/L，β_2_微球蛋白2.61 mg/L。血清游离轻链比值205.26。全身磁共振检查示：全身诸骨于DWI序列呈不均匀稍高信号，左侧第7肋结节（1个）及股骨小结节信号，考虑肿瘤低负荷状态。结合以上检查结果，患者不存在“CRAB”症状（血钙增高、肾功能损害、贫血、骨病）。Durie‑Salmon（DS）分期体系ⅠA，国际分期系统（ISS）Ⅰ期，修订版国际分期系统（R-ISS）Ⅱ期，二次修订版国际分期系统（R2-ISS）Ⅲ期。骨髓标本通过CD138免疫磁珠分选浆细胞后进行FISH检测，结果显示IGH重排阳性，IGH::FGFR3和IGH::CCND1重排阳性（图1），伴1q21扩增（阳性率96.5％）及13q缺失（阳性率91％），根据梅奥MM风险分层与治疗推荐指南4.0（mSMART 4.0）标准判定为高危组。参照美国国立综合癌症网络（NCCN）及国际骨髓瘤工作组（IMWG）的指南[4]，患者确诊为SMM。

**图1:**
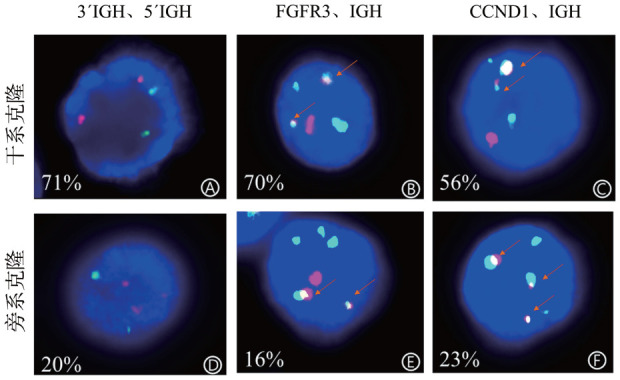
荧光原位杂交检测例1骨髓瘤间期细胞IGH（A、D）、IGH::FGFR3（B、E）、IGH::CCND1（C、F）基因重排 **注** 左下角百分比为信号百分率；IGH双色分离探针：3′IGH基因标记为红色，5′IGH基因标记为绿色；IGH/FGFR3双色双融合探针：FGFR3基因标记为红色，IGH基因标记为绿色；IGH/CCND1双色双融合探针：CCND1基因标记为红色，IGH基因标记为绿色；黄色箭头表示融合基因信号

例2，男，50岁，主因“右侧肋骨疼痛3个月”于2021年11月就诊于我院。患者2021年8月无明显诱因出现右侧肋骨疼痛。就诊于当地医院，胸片示右侧肋骨占位（具体不详）。2021年11月于外院行PET-CT检查示：右侧第2、6侧肋，右侧第9后肋，第10、11胸椎椎体，第4、5腰椎椎体，骶1椎体左侧横突，双侧髂骨，左侧骶骨翼，左侧股骨近端及双侧股骨上段髓腔内见异常放射性核素浓聚，CT见双侧股骨上段髓腔内密度略增高，余骨质呈溶骨性破坏，考虑恶性病变（骨髓瘤）可能。为明确诊断于我院就诊。患者入院后完善相关检查，IgG κ型M蛋白负荷显著升高（血清M蛋白32.07 g/L，尿M蛋白3 180 mg/24 h），骨髓浆细胞广泛浸润（骨髓涂片53％，骨髓活检40％～50％），外周血检出异常浆细胞（9％）。HGB 120 g/L，白蛋白36.4 g/L，肌酐74.8 µmol/L，血钙2.28 mmol/L，乳酸脱氢酶164.2 U/L，β_2_微球蛋白3.33 mg/L，血清游离轻链比值228.91，伴溶骨性骨病。DS分期ⅢA，ISS分期Ⅰ期，R-ISS分期Ⅱ期，R2-ISS分期Ⅲ期。FISH检测提示克隆异质性：93％浆细胞携带IGH::FGFR3单易位，仅5％亚克隆合并IGH::CCND1（图2，图3A、B），同时存在17p缺失（阳性率96％）及13q缺失（阳性率94.5％），依据mSMART 4.0标准判定为高危组。靶向DNA二代测序（NGS）的免疫球蛋白/T细胞受体基因融合分析显示IGH::FGFR3，IGH断裂点位于IGHJ6上游约4 kb处的IGH恒定区，FGFR3断裂点位于该基因下游约101 kb处（图3C）。

**图2:**
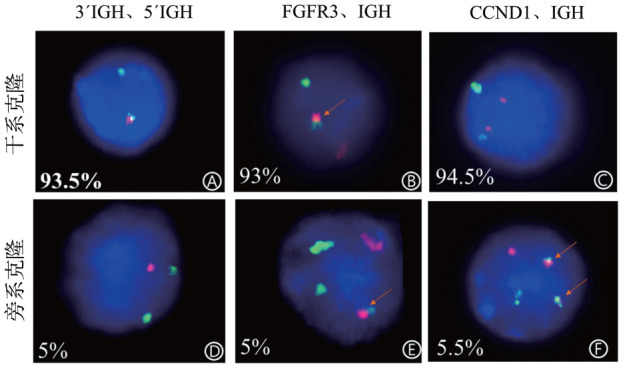
荧光原位杂交检测例2骨髓瘤间期细胞IGH（A、D）、IGH::FGFR3（B、E）、IGH::CCND1（C、F）基因重排 **注** 左下角百分比为信号百分率；IGH双色分离探针：3′IGH基因标记为红色，5′IGH基因标记为绿色；IGH/FGFR3双色双融合探针：FGFR3基因标记为红色，IGH基因标记为绿色；IGH/CCND1双色双融合探针：CCND1基因标记为红色，IGH基因标记为绿色；黄色箭头表示融合基因信号

**图3:**
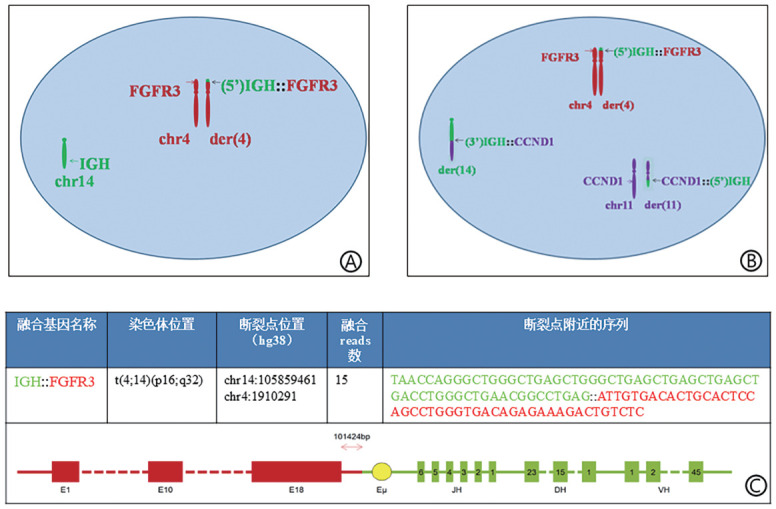
例2干系克隆（A）、旁系克隆（B）示意图及靶向DNA二代测序的免疫球蛋白/T细胞受体基因融合分析（C）结果

参照NCCN及IMWG的指南[4]，例2确诊为MM。采用VRDD方案（硼替佐米2.6 mg，第1、4、8、11天；来那度胺25 mg，第1～14天；地塞米松20 mg，第1、2、4、5、8、9、11、12天；多柔比星脂质体20 mg第3天、60 mg第4天）+达雷妥尤单抗，以及VDD方案（硼替佐米2.6 mg，第1、4、8、11天；地塞米松20 mg，第1、2、4、5、8、9、11、12天；多柔比星脂质体40 mg，第2、3天）+达雷妥尤单抗化疗，患者经过5个疗程的治疗后出院，患者的治疗方案及实验室检查结果见表1。参照2016 IMWG疗效标准[5]，患者疗效评估为非常好的部分缓解。

**表1:** 例2的治疗方案及实验室检查结果

化疗疗程数	化疗方案	血M蛋白（g/L）	血IEF	尿IEF	血FLC差值（mg/L）	FLC比值	尿M蛋白（mg/24 h）	骨髓涂片浆细胞比例	骨髓活检浆细胞比例	外周血涂片浆细胞比例
0	–	32.0	IgG κ	κ	1 296.81	228.9	3180	53％	40％～50％	9％
1	VRDD+dara	–	–	–	–	–	–	–	–	–
2	VRDD+dara	5.2	IgG κ	未见	−1.12	0.88	0	–	–	–
3	VRDD+dara	3.2	IgG κ	未见	−1.82	0.76	0	0	0	0
4	VDD+dara	0	IgG κ	未见	0.90	1.18	0	–	–	–
5	VDD+dara	0	IgG κ	–	0.48	0.92	0	0	0	0

**注** IEF：免疫固定电泳；FLC：游离轻链；VRDD：硼替佐米+来那度胺+地塞米松+多柔比星脂质体；dara：达雷妥尤单抗；VDD：硼替佐米+地塞米松+多柔比星脂质体；–：无数据

## 讨论及文献复习

MM患者中浆细胞IGH双等位基因易位罕见。Tang等[6]在1 087例浆细胞肿瘤患者的FISH检测结果中尚未发现t（11;14）、t（4;14）或t（14;16）易位同时存在，因此作者认为原发性IGH重排遗传事件具有互斥性。本文回顾性分析了既往携带双等位IGH原发性易位MM患者的病例报道（表2），结果显示，1个原发性IGH重排并不排除额外IGH重排的可能。Sáez等[7]通过多色FISH技术发现1例MM患者同时携带IGH::FGFR3/MMSET与IGH::CCND1双易位。Ravindran等[8]报道，1例MM患者的FISH检测显示IGH基因双等位易位导致IGH::FGFR3和IGH::MAF双融合。患者接受后纵隔放疗及6个周期VRD方案（硼替佐米+来那度胺+地塞米松）治疗后，随访12个月未进展。Meseha等[9]报道了1例κ轻链型MM，其遗传学演化呈现显著时空异质性，初诊时仅存在IGH::CCND1单克隆（阳性率93％），经多重治疗抵抗及克隆演化后进展为携带IGH::MAF新发易位的亚克隆，最终形成IGH::CCND1（74％）和IGH::MAF（89％）双等位基因共存状态，患者桥接CAR-T细胞治疗后生存期达6年。IGH::MAF的出现可能源于治疗压力下预先存在的亚克隆扩增或再激活[12]，凸显了动态遗传学监测在优化治疗决策中的价值。Lu等[10]报道了1例罕见的三重打击超高危MM患者携带IGH::FGFR3、IGH::MAF和1q21+，患者经过4个疗程VRD方案治疗后实现了完全缓解，随后在短期内接受了自体造血干细胞移植，随后接受了IRD方案（伊沙佐米+来那度胺+地塞米松）巩固治疗和IR方案（伊沙佐米+来那度胺）维持治疗，目前病情稳定。Baysal等[11]报道了2例包含TP53缺失、IGH::FGFR3伴随IGH::MAF或IGH::MAFB的三重打击MM病例。此外，作为MM病程晚期的遗传学事件，MYC重排也会涉及IGH基因座，当合并原发性IGH重排事件时，即会造成IGH双等位基因易位的发生[13]。

**表2:** 携带双等位IGH原发性易位的多发性骨髓瘤病例

文献来源	性别	年龄	双等位IGH易位	治疗方案	预后
Sáez等[7]	–	–	IGH::FGFR3/MMSET和IGH::CCND1	–	–
Ravindran等[8]	女	46岁	IGH::FGFR3/MMSET和IGH::MAF	纵隔放疗及6个周期VRD方案治疗	1年后未进展
Meseha等[9]	男	63岁	IGH::CCND1和IGH::MAF	嵌合抗原受体T细胞治疗	6年后死亡
Lu等[10]	女	53岁	IGH::FGFR3和IGH::MAFB	4个疗程VRD方案后完全缓解；自体造血干细胞移植；4个疗程IRD方案巩固治疗；IR方案维持治疗	病情稳定
Baysal等[11]	–	–	IGH::FGFR3和IGH::MAF	–	–
Baysal等[11]	–	–	IGH::FGFR3和IGH::MAFB	–	–

**注** VRD：硼替佐米+来那度胺+地塞米松；IRD：伊沙佐米+来那度胺+地塞米松；IR：伊沙佐米+来那度胺；–：未知

本研究提示初发克隆可能携带双重IGH原发性易位事件。在MM中，FGFR3信号传导已被证实可介导STAT3和MAPK的磷酸化，并在IL-6依赖性浆细胞瘤细胞系中与IL-6信号产生协同作用，维持细胞系的增殖与存活[14]，而IGH::CCND1易位可能通过改变抗凋亡通路促进浆细胞存活[15]，双重致癌易位驱动的协同效应可能带来比单个IGH基因易位更差的结局。鉴于双等位IGH易位的罕见性，本研究虽观察到例2对VRDD/VDD方案+达雷妥尤单抗反应良好，但受限于样本量，目前无法提出针对性的新治疗策略。未来需通过多组学整合分析和病例共享建立基于证据的治疗策略。

本研究中，FISH IGH双色分离探针检测揭示了2例MM患者的克隆异质性：例1中IGH双等位基因重排存在于干系克隆；例2中双等位基因重排仅存在旁系克隆（由干系克隆衍生的亚克隆）中。干系克隆携带驱动疾病发生的关键基因组改变，其持续存在是疾病复发的独立危险因素。旁系克隆检测主要用于监测肿瘤演化。通过动态监测干系与旁系克隆的比例变化可早期预警疾病进展。因此，双等位基因易位的平衡是否会持续、克隆大小是否有变化、是否会产生新的亚克隆都需要进一步观察。

为更好地理解双重易位背后详细的分子结构重排，对例2进行了DNA NGS免疫球蛋白融合检测，结果显示IGH::FGFR3融合，导致FGFR3接受IGH Eµ增强子调控，导致其过量表达。值得注意的是，由于送检骨髓标本未经CD138磁珠分选纯化，且IGH::CCND1亚克隆仅占5％（低于NGS检测下限），导致该IGH融合未被检出。提示NGS检测需结合细胞分选以提高低频克隆检出率；单一检测技术可能存在假阴性风险，建议联合FISH与靶向测序互补验证。

综上所述，本研究报道了中国MM患者中IGH双等位基因易位导致IGH::FGFR3与IGH::CCND1易位共存的罕见遗传学事件。鉴于MM的克隆异质性和基因组的高度不稳定性，应用FISH连续检测追踪克隆演变有助于个体化治疗策略的制定。本研究样本量有限且随访时间较短，未来研究应关注双等位基因易位的时空演化规律，同时通过多中心队列研究验证克隆演化与临床结局的相关性。
